# Does subjective sleep quality improve by a walking intervention? A real-world study in a Japanese workplace

**DOI:** 10.1136/bmjopen-2016-011055

**Published:** 2016-10-24

**Authors:** Hikaru Hori, Atsuko Ikenouchi-Sugita, Reiji Yoshimura, Jun Nakamura

**Affiliations:** Department of Psychiatry, University of Occupational and Environmental Health Iseigaoka, Yahatanishi-ku, Kitakyushu, Japan

**Keywords:** Walking, Sleep, PSQI, exercise habits

## Abstract

**Objectives:**

The purpose of this study was to evaluate the impact of a 4-week walking intervention on subjective sleep quality.

**Design:**

A prospective open-label study.

**Participants:**

A total of 490 healthy workers were included in the study. The 490 participants were divided into a group of 214 participants with exercise habits (exercising group, EG) and a group of 276 participants without exercise habits (non-EG).

**Interventions:**

A walking intervention with a target of walking 10 000 steps daily for 4 weeks.

**Outcome measures:**

The Pittsburgh Sleep Quality Index (PSQI) questionnaire was administered twice (before the start and after the end of the study).

**Results:**

Overall, the walking intervention improved the participants’ PSQI global score, sleep latency (minutes), sleep duration (hours), perceived sleep quality factor and daily disturbance factor. Among the EG participants, the walking intervention significantly improved the PSQI global score and perceived sleep quality. Among the non-EG participants, the walking intervention significantly improved the PSQI global score, sleep latency, sleep duration and perceived sleep quality.

**Conclusions:**

A walking intervention might reduce the sleep latency and increase total sleep duration in working persons without exercise habits.

**Strengths and limitations of this study**

- This study is the first study of a walking intervention with a large sample size.
- Although no significant change was observed in the exercising group (EG) after the walking intervention, sleep latency and sleep duration in the non-EG improved significantly.
- Regardless of participants' previous exercise habits, the walking intervention improved perceived sleep quality and total Pittsburgh Sleep Quality Index (PSQI) score as demonstrated by an analysis using the PSQI three-factor model.
- The absence of a control group is a significant limitation, in addition to the lack of an objective measure of self-reported sleep, such as the inclusion of actigraphy.

## Introduction

Chronic sleep deficiency is known to cause mental deterioration, such as daytime sleepiness, impaired motivation and memory decline, as well as to substantially affect the secretion of endogenous hormones and autonomic functioning. People with chronic insomnia have been shown to be susceptible to lifestyle-related diseases such as diabetes mellitus^1^ ^2^ and hypertension.^3–5^ Moreover, patients with lifestyle-related diseases frequently suffer from sleep apnoea^6^ ^7^ and insomnia. Sleep disturbance is known to be an antecedent of and potential risk factor for both depression and suicide.^8–15^

In this study, we examined the effects of walking 10 000 steps per day on the subjective sleep quality of workers and compared these effects in people with and without pre-existing exercise habits.

## Methods

We explained the effects of walking on physical and mental wellness and described the 4-week walking intervention through meetings and leaflets presented to 1298 workers.

This 4-week study sought to evaluate the safety and effect of walking on subjective sleep quality in healthy workers. The study consisted of a screening phase (14 days) and an intervention phase (28 days). In the screening phase, the health status of all participants was screened by a physician. To exclude those with psychiatric disorders, we used the Structured Clinical Interview for DSM-IV-TR (Diagnostic and Statistical Manual of Mental Disorders, Fourth Edition, Text Revision) Disorders (SCID) to screen all participants. None of the participants had a history of neurological, somatic or psychiatric illnesses known to influence sleep disturbance. During the screening phase, the participants wore pedometers and did not change their lifestyles.

We implemented a walking intervention for 490 healthy working people (415 men and 75 women; aged 44.7±10.9 years) (table 1), who were given a target of walking 10 000 steps daily for 4 weeks. Given the changes in the participants' physical health and daily lifestyles, walking 10 000 steps was set as a non-binding target. We recommended 10 000 steps of walking per day without regulating either the distance walked or the speed of walking. In this study, the target was set at 10 000 steps because expending at least 2000 kcal of energy per week (∼300 kcal/day) through physical activity is recommended, and individuals can achieve this target by walking ∼10 000 steps per day. All participants used a pedometer. In addition, the 490 walking participants were divided into a group of 214 participants with existing exercise habits (the exercising group (EG)) and a group of 276 participants without exercise habits (the non-EG). The participants in the EG had been exercising for 30 min or more per day, two or more times per week, for at least 1-year.

**Table 1:** Demographics of the study participants

	Total	EG	Non-EG
Sex (M/F)	415/75	178/36	234/42
Age (years)	44.41±0.55	44.02±0.41	44.69±0.91

EG, exercising group; F, female; M, male.

We sent a reminder email to the participants on Monday of each week to check on the participant's physical status and continue 10 000 steps walking per day.

Pedometer data were collected for weeks 2 and 4.

The Pittsburgh Sleep Quality Index (PSQI) questionnaire was administered twice (before the study began and after its completion).

### Pittsburgh Sleep Quality Index

The PSQI is a 19-item self-reported instrument designed to measure a person's sleep quality and sleep patterns. The index contains seven domains: sleep duration, sleep syndrome, sleep latency, daytime dysfunction caused by sleepiness, sleep efficacy, overall sleep quality and use of sleep medications.^16^ Each domain is scored from 0 to 3, with higher values indicating poorer sleep quality and a clinical cut-off point of 5. A total of 63 (12.9%) participants were identified as poor sleepers (PSQI global >5) in this study. All participants were required to complete the Japanese version of the PSQI to report their self-rated sleep quality over the past month.^16–19^ Moreover, we used PSQI three-factor scoring:^20^ sleep efficiency (sum of sleep duration and habitual sleep efficiency components), perceived sleep quality (sum of subjective sleep quality, sleep latency and sleeping medication use components) and daily disturbances (sum of sleep disturbance and daytime dysfunction components). We also analysed the raw values of sleep latency and sleep duration from the PSQI.

### Statistics

The data are reported as the means±SDs. Analyses of variance with repeated measures were used to compare the sleep parameters at baseline (before the walking intervention) to the parameters after the walking sessions.

To compare the baseline characteristics of the PSQI scores, this study used Student's t-test to assess age and sex differences between the EG and non-EG. A paired Student's t-test was also used to examine differences in the PSQI scores before and after the walking intervention. Missing values were completely random; therefore, a mean was substituted for a missing value in this analysis. The relationship between the changes in steps/day and the changes in subjective sleep quality was examined using Pearson's correlation coefficients.

A value of p<0.05 was considered to be statistically significant.

## Results

**Figure 1:**
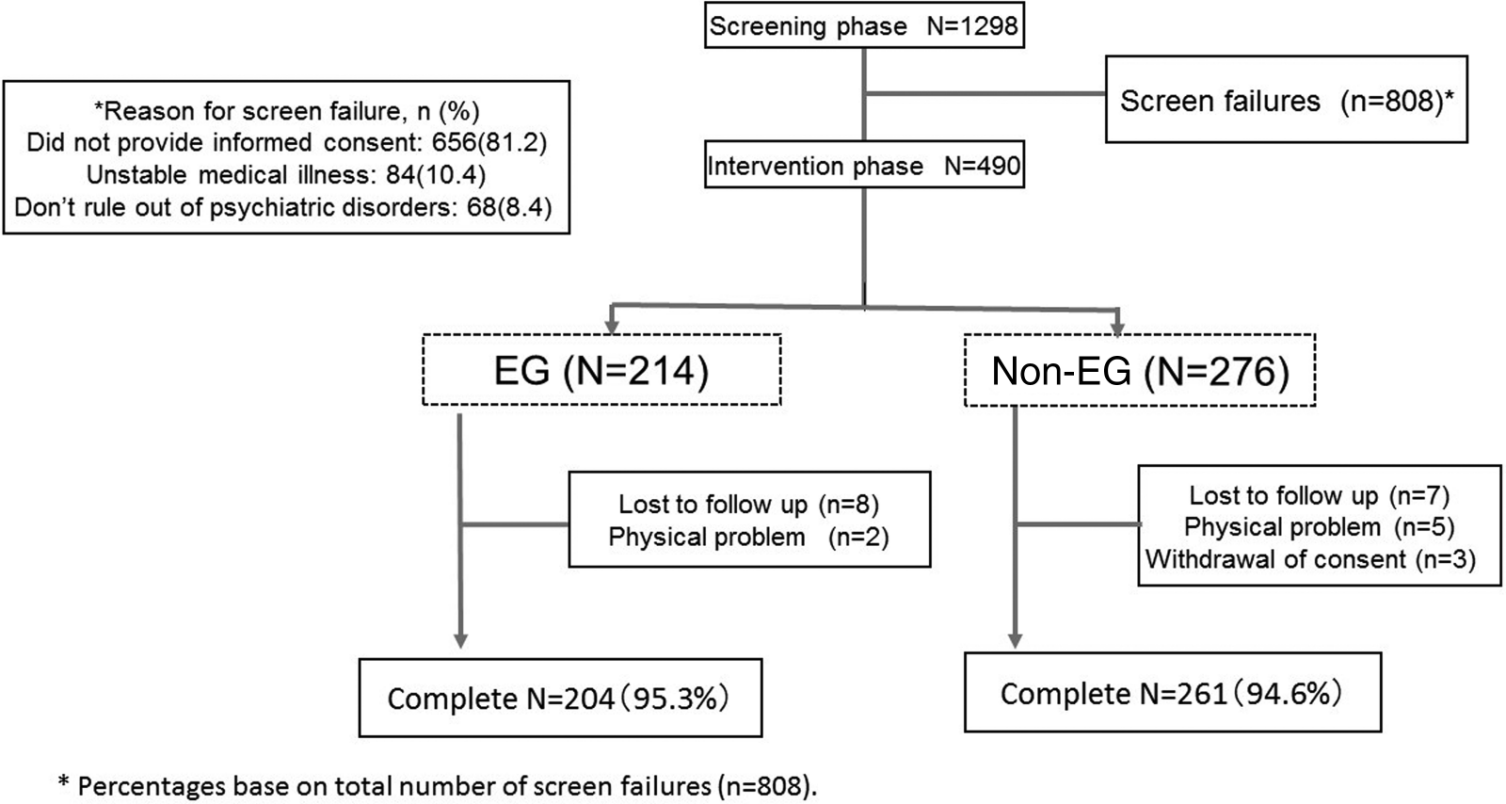
The study protocol. EG, exercising group.

The protocol is shown in figure 1. We explained the effects of walking on physical and mental wellness and described the 4-week walking intervention through meetings and leaflets presented to 1298 workers. Of these 1298 participants, 808 did not wish to participate, and the remaining 490 (415 males and 75 females; aged 44.7±10.9 years) agreed to participate in the walking intervention.

**Figure 2:**
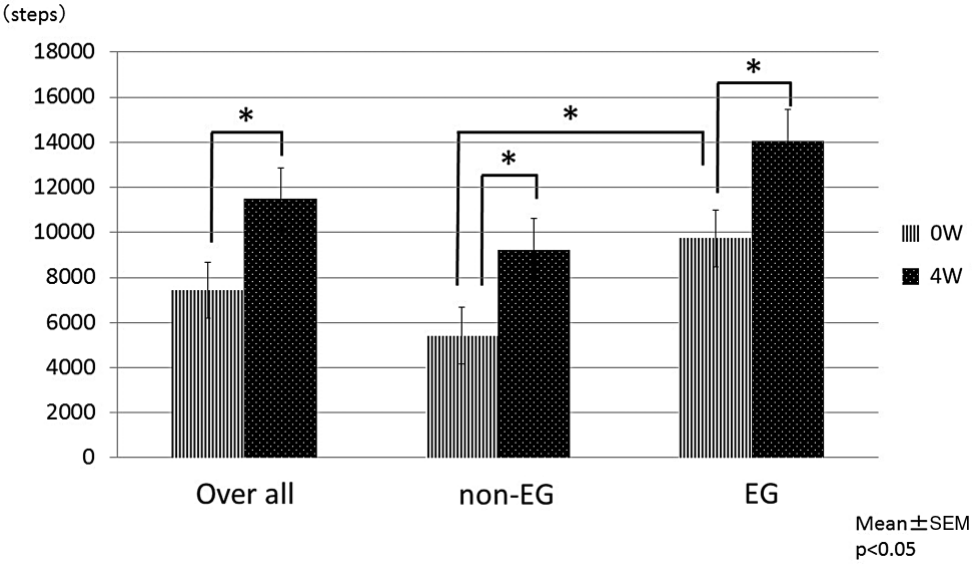
The number of steps 0 and 4 weeks. EG, exercising group; W, week. *p<0.05.

Although the number of steps before the start of the study was significantly greater in the EG participants than in the non-EG participants (EG: 9739.1±2826.2 steps, non-EG: 5415.0±2787.2 steps (p value 0.01)), the number increased significantly in both groups after the walking intervention (EG: 14048.0±3982.6 steps (p<0.01) compared with the number before the walking intervention), non-EG: 9223.9±2932.7 steps (p<0.01) compared with the number before the walking intervention) (figure 2).

The participants' baseline sleep status is presented in table 2. The EG and non-EG participants differed significantly in their sleep duration (hours) (p<0.01) and sleep efficiency factor (p=0.02) scores at baseline. However, the results revealed no significant differences between the groups' PSQI global score (p=0.051), sleep latency (minutes) (p=0.25), perceived sleep quality factor (p=0.89) and daily disturbance factor (p=0.18).

**Table 2:** Effects of walking on subjective sleep quality

	Total 0 week	Total 4 weeks	p Value	EG 0 week	EG 4 weeks	p Value	non-EG 0 week	non-EG 4 weeks	p Value	p Value (EG 0 week vs non-EG 4 weeks)
PSQI global score	3.8±2.2	3.3±2.0	<0.01	3.6±2.0	3.3±2.0	<0.01	4.0±2.4	3.4±2.1	<0.01	0.051
Sleep latency (min)	19.3±15.7	15.4±15.1	<0.01	18.4±13.7	17.5±16.3	0.50	20.2±17.4	13.7±13.9	<0.01	0.25
Sleep duration (hours)	6.1±0.9	6.2±1.0	<0.01	6.2±0.9	6.3±0.9	0.10	6.0±0.8	6.1±1.0	0.01	<0.01
PSQI global score >5 (%)	12.9%	10.4%	0.23	10.3%	8.4%	0.51	14.9%	12.0%	0.31	0.12
Sleep efficiency	1.2±0.9	1.1±0.9	0.056	1.1±0.9	1.0±0.9	0.42	1.3±0.9	1.1±0.9	0.29	0.02
Perceived sleep quality	1.5±1.0	1.3±1.0	<0.01	1.5±1.0	1.3±1.0	0.03	1.5±1.1	1.2±1.0	0.02	0.89
Daily disturbances	1.1±0.6	1.0±0.6	0.02	1.0±0.6	1.0±0.6	0.18	1.1±0.6	1.1±0.6	0.32	0.18

EG, exercising group; PSQI, Pittsburgh Sleep Quality Index.

Moreover, the 4-week walking intervention improved the PSQI global score (p<0.01), sleep latency (p<0.01), sleep duration (p<0.01), perceived sleep quality factor (p<0.01) and daily disturbance factor (p=0.02) for all participants; the PSQI global score (p<0.01) and perceived sleep quality factor (p=0.03) for the EG; and the PSQI global score (p<0.01), sleep latency factor (p<0.01), sleep duration (p=0.01) and perceived sleep quality factor (p=0.02) for the non-EG.

Sleep latency decreased significantly (week 0:19.3±15.7 min, week 4: 15.4±15.1 min, p<0.01), whereas sleep duration increased significantly (week 0: 6.1±0.9 hour, week 4: 6.2±1.0 hour, p<0.01).

The results revealed a negative correlation between changes in mean steps (before and 4 weeks after the walking intervention) and changes in perceived sleep quality scores (r=−0.19, p<0.05) and sleep latency scores (r=−0.22, p<0.05) (table 3).

**Table 3:** Association between changes in mean steps (before and after the 4-week walking intervention) and changes in the raw data (sleep latency (minutes), sleep duration (hours)) and Pittsburgh Sleep Quality Index (PSQI) three-factor scores (before and after the 4-week walking intervention)

	⊿Raw data	⊿Steps (4–0 weeks)
⊿PSQI three-factor score (4–0 weeks)	Sleep latency	−0.22*
	Sleep duration	−0.13
	Sleep efficiency	−0.04
	Perceived sleep quality	−0.19*
	Daily disturbance	−0.02

*p<0.05.

## Discussion

The results of our study showed that the walking intervention reduced the time to sleep onset and caused a subjective increase in the total sleep duration for working people without exercise habits. This finding suggests that physical exercise may improve sleep quality in this population. In our study, healthy workers without exercise habits (non-EG) exhibited lower sleep efficiency PSQI scores and lower sleep duration than did the participants with exercise habits (EG) at baseline. The 4-week walking intervention was effective in improving the PSQI global score, sleep duration and sleep latency of the non-EG participants. Moreover, for both the EG and non-EG, the exercise intervention proved to be effective in improving PSQI global scores. Inspection of the separate items of the sleep quality scale revealed that perceived sleep quality in particular improved among participants in the exercise condition. Contrary to expectations, we did not find an effect of exercise on sleep latency and sleep duration in the EG. These results may be explained by the fact that most of the EG participants were already sleeping well before the intervention; hence, potential for improvement was limited because regular exercise had already improved their sleep latency and sleep duration.

Kredlow *et al*^21^ reported a meta-analysis showing that acute exercise has small beneficial effects on total sleep time, sleep onset latency, sleep efficiency, stage 1 sleep and slow wave sleep; a moderate beneficial effect on wake time after sleep onset; and a small effect on rapid eye movement sleep. Youngstedt *et al*^22^ reported a meta-analysis showing that acute exercise increased total sleep time. Another study found that exercise is also associated with decreases in light-sleep stages and increases in the deeper, more restorative stages of sleep.^23^ These findings suggest that both acute and chronic exercise have beneficial effects on sleep.

Habitual exercise has been associated with better sleep conditions in the general population, and exercise programmes have been shown to improve the self-rated sleep quality of the elderly.^24–26^

In addition, sleep disturbances can be a risk factor for various lifestyle-related diseases. Exercise therapy, including walking, is known to be useful in preventing and treating lifestyle-related diseases. In other words, exercise therapy can prevent lifestyle-related diseases, as well as alleviate depression and sleep disturbances. Previous longitudinal epidemiological studies have shown that persistent insomnia over time is associated with a higher incidence of depressive disorders.^8–10^ ^11^ ^13^ Another previous study explored the evolution of insomnia over time in association with several physical health problems and depressive mood.^27^

This study has some limitations. First, the study participants were healthy Japanese workers in their 20s to 60s. Therefore, the ability to generalise the results of this research to unhealthy men and women and to other ethnic/racial and minority groups is limited. Second, the absence of a control group is also a significant limitation of this study. Third, the outcomes were based on self-reported data rather than objective measures such as actigraphy and polysomnography.

In conclusion, our study revealed that a walking intervention may reduce the time to sleep onset and increase total sleep duration in working people without exercise habits. In the future, we plan to conduct a randomised control trial comparing a group with a walking intervention with a control group with no intervention based on objective measures such as actigraphy and polysomnography.
